# Biopesticide transplant dips and foliar acaricide applications for control of cyclamen mite (*Phytonemus pallidus*) in strawberry

**DOI:** 10.1007/s10493-024-00974-9

**Published:** 2025-01-17

**Authors:** Justin M. Renkema

**Affiliations:** Agriculture and Agri-Food Canada, London Research and Development Centre–Vineland Campus, Vineland Station, ON Canada

**Keywords:** Fenazaquin, Pyridaben, Chlorfenapyr, Spiromesifen, Abamectin

## Abstract

Cyclamen mite (*Phytonemus pallidus*) causes injury to new growth of strawberry plants and is difficult to control because it is protected by folded leaves and plant crowns. Since cyclamen mite is easily transferred from strawberry nurseries to fruiting fields, dipping transplants in biopesticides may reduce initial populations. However, cyclamen mite numbers at 1 and 3 months-after-planting, and yield and cyclamen mite injury to fruit in the following season did not differ among transplants immersed for 30 s in Captiva® Prime, EcoTrol® EC, Landscape Oil, SuffOil-X® or Kopa Insecticidal Soap or the untreated control. Cyclamen mite is primarily controlled with foliar applications of acaricides, but there are few registered products. In greenhouse experiments, fenazaquin and pyridaben reduced cyclamen mite numbers by more than 90% in new leaves compared to the control, similar to that of the standard abamectin. New leaf injury ratings were reduced from 1 on average (scale of 0–3; 0 = no injury) pre-application to 0.25–0.5 for fenazaquin, pyridaben, and abamectin-treated plants compared to increasing to 2 for control plants 2 weeks after application. Spiromesifen and chlorfenapyr reduced cyclamen mite numbers in folded leaves in one greenhouse experiment. In the field, all acaricides reduced cyclamen mite numbers by 90–99% at 2- and 6-weeks post-application and by 75–90% at 10 months post-application. Abamectin and pyridaben resulted in 0.5–1.0% of strawberries with cyclamen mite damage compared to 3.0% for the control. All acaricides except chlorfenapyr improved strawberry yield and size. Overall, fenazaquin, pyridaben and spiromesifen should help diversify the chemical toolbox for cyclamen mite in field strawberry.

## Introduction

Cyclamen mite (*Phytonemus pallidus*) is a widespread pest of cultivated strawberry and ornamentals, including cyclamen, chrysanthemum, gerbera, and African violet (Denmark 2017). Adults and nymphs inhabit concealed spaces among new, folded leaves or flower buds, and a proportion of a population is in the plant crown (Fitzgerald et al. 2008). Injury occurs from mite feeding, and in strawberry symptoms appear first as mild leaf curling and discoloration and can progress to extensive plant stunting, leaf browning and small, seedy fruit if populations are not controlled (Alford 1972; Fitzgerald et al. 2008). Cyclamen mite is small (adult female: 0.25 mm) and difficult to detect at low population levels, and acaricides are relied on for prophylactic control or applied once plant injury is observed.

Endosulfan was relied on for many decades in strawberry nurseries for cyclamen mite and other pest control (Schaefers 1963). Since the phase-out of endosulfan in 2016 in North America and earlier in European countries (Health Canada 2011), steam and controlled atmosphere temperature treatments have been tested and shown to be effective for nearly eliminating cyclamen mite on transplants (van Kruistum et al. 2015; Johansen et al. 2022; Bernier et al. 2023; Pate et al. 2024). However, such treatments require investment in specific equipment, and a simpler pre-plant treatment may be more feasible in some scenarios. In floriculture, biopesticide dips reduced silverleaf whitefly (*Bemisia tabaci*) and Western flower thrips (*Frankliniella occidentalis*) on poinsettia cuttings, and horticultural oil dips controlled two-spotted spider mite (*Tetranychus urticae*) on multiple ornamental crops (Buitenhuis et al. 2016, 2019; R. Buitenhuis, personal communication). In strawberry, pre-plant dip treatments in fungicides for control of crown rot caused by anthracnose (*Colletotrichum acutatum*) have become standard in some regions (Turechek et al. 2006; Haack et al. 2018), but dips have not been tested against cyclamen mite.

Abamectin (Agri-Mek® SC) is the only acaricide registered for cyclamen mite in Canada in strawberry. It has a proven record against cyclamen mite, providing high levels of control (90–100%) when applied with a surfactant (Zalom et al. 2009; Fountain et al. 2010; Łabanowska et al. 2015). Abamectin is translaminar in apples and pears (Beers et al. 1997) and strawberry (Walsh et al. 1996), a property that likely contributes to its efficacy against cyclamen mite. Canola oil (Vegol® Crop Oil) is also registered for cyclamen mite in strawberry, but as a stand-alone it was not effective in the laboratory or in the field after 2 foliar applications post-renovation in matted-row strawberry (Spooner-Hart and Herron 2003; Lyle 2022).

Fenazaquin (Magister® SC) was recently registered in Canada in strawberry for two-spotted spider mite (Health Canada 2023). Fenazaquin was first commercialized in 1993 and has a history of use in tree fruit in Europe against spider mites and European red mite (*Panonychus ulmi*) (Longhurst et al. 1992; Solomon et al. 1993; DeKeyser 2005; Urbaneja et al. 2008). It is also effective against other Tetranychidae, such as southern red mite (*Oligonychus ilicis*) in blueberry (Lopez and Liburd 2020). For non-tetranychid mite pests, fenazaquin was not effective against the apple rust mite (*Aculus schlechtendali*) (Eriophyidae). However, it provided moderate to excellent control of the coconut mite (*Aceria guerreronis*) in coconut and broad mite (*Hemitarsonemus* = *Polyphagotarsonemus latus*) (Tarsonemidae) in chilli and sesame (Walunj and Pawar 2000; Pushpa and Nandihalli 2008; Sarkar et al. 2013; Bathani et al. 2019). In one experiment in 1995, Łabanowska (2003) found 94 and 67% fewer mites 7 and 21 days, respectively, after two applications of fenazaquin (formulated as Magus 200® SC) compared to the control.

Since the phase-out of endosulfan, at least 13 acaricides have been tested, and those with a group 6, 21 or 23A mode-of-action (IRAC 2024) typically provided good control of cyclamen mite in strawberry (Łabanowska 2006a,b; Zalom et al. 2009; Fountain et al. 2010; Łabanowska et al. 2015). Pyridaben reduced cyclamen mite populations by > 80% in field experiments (Łabanowska et al. 2015), and spiromesifen was moderately effective in the laboratory but ineffective in one experiment in the field (Zalom et al. 2009; Fountain et al. 2010). Pyridaben (Nexter® SC) and spiromesifen (Oberon™ Flowable) are registered in Canada in field strawberry for two-spotted spider mite. Chlorfenapyr is a group 13 mode-of-action insecticide/acaricide not previously tested against cyclamen mite but with efficacy against broad mite (Sarkar et al. 2013; Halder et al. 2015). Chlorfenapyr (Pylon®) is registered for use in greenhouses in Canada for mite control in some vegetables and could be useful in the burgeoning greenhouse strawberry industry, where there are currently no registered acaricides.

Since reliance on one acaricide, abamectin, is unsustainable, the goal of the research was to diversify the chemical toolbox for cyclamen mite. The specific objectives were to determine the effect of pre-plant biopesticide dips on cyclamen mite in strawberry transplants and the effect of post-plant foliar-applied acaricides on cyclamen mite on strawberry in the greenhouse and field. Biopesticides selected are registered in strawberry or other horticultural crops in Canada, and if efficacious could help reduce foliar acaricide applications, particularly in the planting year. The outcome of the acaricide experiments is new information on the efficacy of additional products for cyclamen mite.

## Materials and methods

### Greenhouse foliar acaricides

Bare-root strawberry transplants were acquired from a nursery in Ontario, Canada. The cultivar ‘Brilliance’ was planted in October 2020 for the winter 2021 experiment, and the cultivar ‘Jewel’ was planted in June 2021 for the winter 2022 experiment. Transplants were placed singly in PRO-MIX BX Mycorrhizae potting mix (PRO-MIX®, Premier Tech, Rivière-du-Loup, QC) in black plastic pots (top diameter = 16 cm, height = 18 cm) in a research greenhouse in Vineland, Ontario. Plants were watered daily and fertilized weekly by-hand (Plant-Prod® 20-20-20 classic, PlantProducts®, Ancaster, ON), and flower buds and runner tips were removed at the start of each experiment.

On 17 February 2021 and 9 February 2022, plants were infested with cyclamen mites from a colony maintained at research facilities in Vineland, Ontario. The colony was started in May 2018 from infested leaves collected from a strawberry field near Jordan, Ontario. The colony was reared on potted strawberry plants (‘Jewel’ and ‘Annapolis’) in a growth chamber at 24 °C, 50–60% RH and a 16 h photoperiod. Young, tightly-wrapped leaves were removed from colony plants, split into leaflets and inspected under magnification to ensure each contained about 30 cyclamen mites. A leaflet was placed into the crown of each strawberry plant using a pair of blunt tweezers.

Acaricides (Table 1) were mixed in distilled water with a non-ionic wetting and spreading agent (0.25% v/v, Agral® 90, Syngenta, ON,) at the highest label rate. An acaricide (2 mL) was applied 10 March 2021 and 3 March 2022 to the crown(s) and young leaves of each plant with an air brush paint sprayer (Paasche H Series, Paasche, WI) attached to a mini air compressor (Paasche Mini Air Compressor model D500SR, Paasche, WI) in a fume hood. Plants were moved back to a greenhouse table and arranged in a completely randomized design with nine replications each year and 50 cm between plants.

**Table 1:** Biopesticides for transplant dips (2021) and acaricides for foliar application (2022) against *Phytonemus pallidus* in strawberry research plots. Products with the same number were applied to the same plots. Control plots (#6) with the surfactant Agral® 90 were the same each year

Plot	Product	Active ingredient(s) (% or conc.)	Manufacturer	Appl. rate
1	Captiva® Prime	capsicum oleoresin extract (7.6), garlic oil (23.4), canola oil (55)	Gowan Co	0.25%
2	EcoTrol® EC	rosemary oil (10), peppermint oil (12)	EcoSMART Technologies Inc	0.25%
3	Kopa Insecticidal Soap	potassium salts of fatty acids (47)	Neudorff North America	1.00%
4	Landscape Oil	mineral oil (99)	Plant Products Inc	0.25%
5	SuffOil-X®	mineral oil (80)	BioWorks Inc	0.25%
1	Agri-Mek® SC	abamectin (84 g/L)	Syngenta Inc	225 mL/ha
2	Magister® SC	fenazaquin (205 g/L)	Gowan Co	2.34 L/ha
3	Nexter® SC	pyridaben (450 g/L)	Gowan Co	500 mL/ha
4	Pylon®	chlorfenapyr (240 g/L)	BASF Canada Inc	300 mL/ha
5	Oberon™ Flowable	spiromesifen (240 g/L)	Bayer CropScience Inc	1.16L/ha

In 2022 only, plants were rated 3 March (prior to acaricide application) and 17 March using a visual scale, where 0 = no damage, green, smooth leaves; 1 = slight damage, slight yellowing of leaf veins and wrinkling of leaves; 2 = moderate damage, yellowing and browning of leaf with significant leaf wrinkling; 3 = severe damage, leaf is small, brown, necrotic and does not open. Plants were destructively sampled 24 March 2021 and 17 March 2022, 2 weeks after miticides were applied. Unfolded, mature leaves were removed and discarded, and all young, folded leaves (1–5 per plant) were placed in a centrifuge tube (50 mL) partially filled with 70% ethanol. Crowns (1–2 per plant) were cut at the soil level and placed in a centrifuge tube with ethanol. Tubes were stored in a refrigerator until a triple-wash-and-rinse method (Renkema et al. 2022) was used to count motiles (adults and larvae) using a stereomicroscope at 20X magnification; eggs were not counted.

### Field biopesticide dips and foliar acaricides

In 2021, 36 tilled research plots at a research farm near Jordan Station, Ontario (43.177, − 79.359) were established in a 63 × 12 m area. Each plot was a single row of 15 plants at 30 cm in-row plant spacing so that the plot was 4.5 m long There were 4 rows of plots with 3 m between rows and 3.5 m between plots within rows. Plots were arranged in a checkerboard pattern with blank space in the row adjacent to each plot. Six replicates of each treatment (Table 1) were arranged in a randomized complete block design, and each block was 2 adjacent rows by 3 plots per row. Tillage, hand-weeding, and glufosinate ammonium (Ignite® SN) were used to maintained weeds in and around plots. Aisles were planted with ryegrass in mid-June as a ground-cover. Plots were sprayed with boscalid + pyroclostrabin (Pristine® WG) on 23 July and mycobutinyl (Nova® 40WP) on 2 August for powdery mildew (*Sphaerotheca macularis*). Plots were not irrigated and received no other fungicides, insecticides, or fertilizers.

Bare-root strawberry transplants (‘Jewel’) were acquired from a nursery in Nova Scotia, Canada in May 2021. On 30 May, transplants were removed from a refrigerator, and 8 out of 15 transplants per plot were infested by placing a strawberry leaflet with 20–30 cyclamen mite motiles from the Vineland laboratory colony into the transplant crown using tweezers. All transplants were placed in bunches of 8 mite-infested or 7 mite-noninfested on plastic trays in a growth chamber at 10 °C and 80–90% relative humidity for 2 days. The roots of the transplants were misted with water 3 times daily.

On 1 June, each biopesticide was prepared at the recommended rate (Table 1) in 6, 2L plastic bottles with distilled water and Agral® 90 (0.25% v/v). Each bottle was shaken by-hand for ~ 1 min and then poured into a white, plastic pail (4L). The 15 transplants per plot were submerged in 2 bunches (8 mite-infested and 7 mite-noninfested) simultaneously by-hand in a biopesticide mixture for 30 s, gently moving the bunches to agitate the mixture. Each set of 15 transplants was submerged in a different pail with a new bottle of biopesticide. Transplant bunches were placed on plastic trays in the shade for 15 min to dry before they were set by-hand in the plots. In each plot, mite-infested and mite-noninfested transplants were alternated.

Survival of each plant in every plot was assessed on 11 June, and dead plants were replaced with new, biopesticide-treated transplants on 14 June. Samples of 5 new, folded leaves were removed at the petiole from 5 randomly-selected of the 8 mite-infested plants per plot on 30 June, and 5 leaves were removed from 5 randomly selected plants per plot on 2 September because the mother plants had runnered and it was difficult to distinguish mother from daughter plants. Leaves were stored and assessed for cyclamen mites as described above for the greenhouse experiments.

In 2022, plots were sprayed with cyprodinil + fludioxinil (Switch 62.5® WG) and folpet (Folpan 80 WDG) on 19 May and folpet again on 31 May for disease control. All ripe strawberries per plot were hand-picked every 3–4 days, and total weight (kg), number of marketable strawberries and number of cyclamen-mite injured strawberries (small, seedy, bronzed) were determined during peak harvest on 20, 24, and 28 June. Averages per harvest were calculated as there were a few missing data points on some days.

After plot renovation on 25 July 25 2022, acaricides were prepared in distilled water and Agral® 90 (0.25% v/v) at the highest label rate to a volume of 1000 L/ha (600 mL per plot). Acaricides were applied on 2 August with a CO_2_-powered backpack sprayer (Model 315FG2, R&D Sprayers, Opelousas, LA) at 2.7 atm with one over-the-top pass using a hand-held wand sprayer with two nozzles (DG TeeJet Drift Guard Flat Spray Tip, TeeJet® Technologies, Glendale Heights, IL). Samples of 5 young, folded leaves were collected from 5 randomly selected plants per plot on 2 August (pre-application), 16 August and 13 September, stored and assessed for cyclamen mites as described above for the greenhouse and biopesticide dip experiments.

In 2023, plots were sprayed with fluopyram + trifoloxystrobin (Luna Sensation) on 9 June and pyrimethanil (Scala SC) on 15 June for disease control. Leaf samples for cyclamen mite assessments were collected 8 June as in 2022. All ripe strawberries per plot were hand-picked on 14, 19, 22, 26, 29 June and 4 July, and the number and weight of marketable, cyclamen mite-injured and other unmarketable strawberries was determined. Average strawberry size (g) per plot per harvest date was determined by dividing the total weight by the total number of strawberries.

### Statistical analysis

For the greenhouse experiments, an analysis of variance (ANOVA) was used to determine if the acaricide affected the numbers of cyclamen mite on the leaves and crowns of plants and the plant rating in 2022. In 2021, one control plant had no cyclamen mite on any of the leaves or crown and was removed from the ANOVAs, and one plant receiving abamectin had no new leaves and was removed from the ANOVA for leaves. For the field experiments, a generalized linear mixed model (ANOVA) with a normal distribution and block as a random effect was used to determine if the acaricide or biopesticide had an effect on the numbers of cyclamen mite on the leaves and the yield metric assessed. Residuals were checked for homoscedasity of error variance; no data transformation was necessary. Tukey’s post hoc HSD test was used to separate means when ANOVAs indicated significant differences. All analyses were conducted in JMP® 17.2.0 at α = 0.05.

## Results

### Greenhouse foliar acaricides

**Fig. 1:**
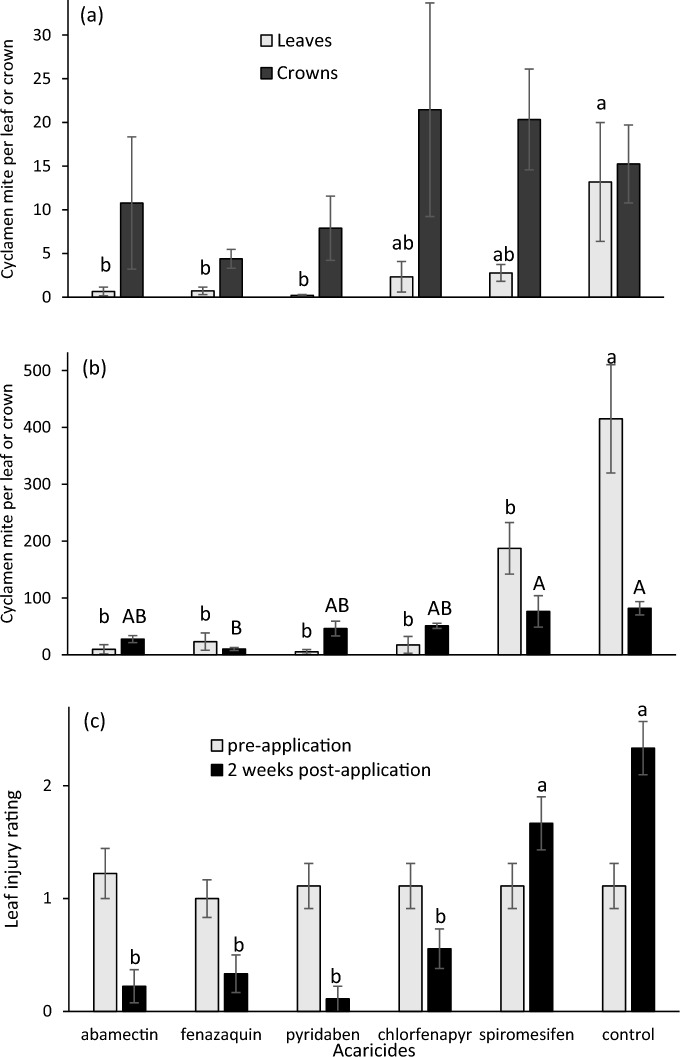
Mean (± SEM) number of *Phytonemus pallidus* motiles (adults, nymphs) in new leaves and crowns of greenhouse strawberry plants two weeks after acaricides were applied in **a** 2021 and **b** 2022, and **c** injury ratings of new leaves (0–3 scale) in 2022. Means of the same plant part with the same letter in each panel are not significantly different (Tukey’s HSD, α = 0.05)

In 2021, there was an effect of acaricide on the number of motile cyclamen mites on new, folded leaves (*F* = 3.15; df = 5, 46; *P* = 0.016) but not in plant crowns (*F* = 1.02; df = 5, 47; *P* = 0.417). There were more mites in control plant leaves than on leaves from plants sprayed with abamectin, fenazaquin or pyridaben (Fig. 1A).

In 2022, there was an effect of acaricide on the number of motile cyclamen mites on new, folded leaves (*F* = 13.99; df = 5, 48; *P* < 0.001) and in plant crowns (*F* = 4.02; df = 5, 48; *P* = 0.004). There were more mites on control plant leaves than on leaves from plants sprayed with any of the acaricides, and there were more mites in control plant crowns and those sprayed with spiromesifen compared to those sprayed with fenazaquin (Fig. 1B). There was no difference in plant ratings for cyclamen mite injury prior to acaricide applications (*F* = 0.12; df = 5, 48; *P* = 0.986), but 2 weeks after application there was an effect of acaricide (*F* = 7.48; df = 5, 48; *P* < 0.001). Control plants and plants sprayed with spiromesifen had more injury than those sprayed with the other acaricides (Fig. 1C).

### Field biopesticide dips and foliar acaricides

There was no effect of dipping transplants in biopesticides on the number of cyclamen mite on new, folded leaves on 30 June or 2 September, approximately 1 and 3 months after dipping transplants, respectively (Table 2). Plant survival 10 days-after-planting was not affected by biopesticide, nor was total yield or the percent of strawberries with cyclamen mite injury in 2022 (Table 2). In addition, the number of marketable strawberries was not affected by biopesticide (*F* = 0.51; df = 5, 25; *P* = 0.766) nor was the strawberry size (*F* = 0.44; df = 5, 25; *P* = 0.820) (data not shown).

**Table 2:** Results of analysis of variance (ANOVA) and mean (± SEM) number of *Phytonemus pallidus* motiles (adults, nymphs) per new, folded leaf, percent survival, total yield and percent cyclamen mite injured fruit from strawberry plants in research plots after mite-infested transplants were dipped in biopesticides prior to planting on 1 June 2021

Biopesticide	No. of *P. pallidus* post-dipping				% plant survival		Total yield (kg)		% cyclamen mite injury	
	29 days		94 days		10 days		20, 24, 28 June 2022			
Captiva® Prime	0.53	(0.16)	31.50	(15.13)	92.2	(3.4)	4.48	(0.26)	3.1	(0.3)
EcoTrol® EC	0.33	(0.19)	18.53	(4.37)	98.7	(1.3)	4.92	(0.35)	5.8	(0.7)
Kopa Soap	0.30	(0.14)	18.47	(6.68)	97.4	(1.6)	4.87	(0.27)	3.6	(0.6)
Landscape Oil	0.33	(0.09)	27.30	(7.39)	91.0	(3.7)	4.57	(0.32)	2.6	(0.7)
SuffOil-X®	0.60	(0.23)	28.47	(8.89)	100.0	(0.0)	4.50	(0.41)	3.3	(1.1)
control	0.63	(0.29)	18.22	(6.90)	96.2	(1.7)	4.39	(0.29)	4.3	(0.6)
*F*	0.65		0.50		2.22		0.45		2.20	
*P*	0.667		0.774		0.083		0.809		0.086	

*df = 5, 25 for each ANOVA

Prior to foliar acaricide application, there was no difference in the number of motile cyclamen mite among plots, but at 2 and 6 weeks and about 10 months post-application, there were effects of acaricides on number of cyclamen mite (Table 3). For all post-application sampling, there were more cyclamen mite in the control plots than in plots sprayed with an acaricide (Table 3).

**Table 3:** Results of analysis of variance (ANOVA) and mean (± SEM) number of *Phytonemus pallidus* motiles (adults, nymphs) per new, folded leaf from strawberry plants in research plots, pre-application and 2 and 6 weeks and 10 months post-application of foliar acaricides on 4 August 2022 at the equivalent of an application volume of 1000L/ha. Means with the same letter in the same column are not significantly different (Tukey’s HSD, α = 0.05)

Acaricide	Pre-application sampling	Post-application sampling					
		2 weeks		6 weeks		10 months	
abamectin	16.50 (10.50)	1.83	(1.18)b	4.07	(3.39)b	32.00	(18.27)b
fenazaquin	12.70 (4.60)	0.20	(0.13)b	4.80	(2.00)b	29.77	(13.81)b
pyridaben	19.10 (6.25)	0.20	(0.09)b	1.07	(0.44)b	14.33	(11.71)b
chlorfenapyr	10.17 (3.78)	0.10	(0.09)b	1.33	(0.52)b	14.67	(6.08)b
spiromesifen	13.37 (3.63)	1.53	(0.56)b	6.57	(1.21)b	23.90	(4.76)b
control	34.37 (13.36)	25.00	(6.84)a	59.53	(14.27)a	134.43	(24.71)a
*F*	1.12	11.70		13.48		10.49	
*P*	0.376	< 0.001		< 0.001		< 0.001	

*df = 5, 25 for each ANOVA

**Fig. 2:**
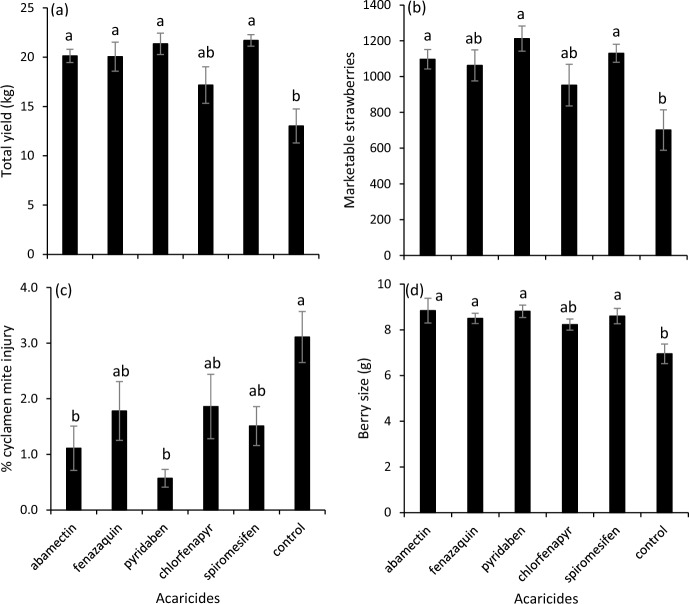
Mean (± SEM) **a** yield of total strawberries, **b** number of marketable strawberries, **c** percent of strawberries with *Phytonemus pallidus* injury, and **d** strawberry size from research plots harvested 14, 19, 22, 26, 29 June and 4 July 2023 when foliar acaricides were applied 4 August 2022. Means with the same letter in the same panel are not significantly different (Tukey’s HSD, α = 0.05)

There was an effect of acaricide application on the total weight (yield) of strawberries harvested (*F* = 7.52; df = 5, 25; *P* < 0.001), the number of marketable strawberries (*F* = 4.53; df = 5, 25; *P* = 0.005), the percent strawberries with cyclamen mite injury (*F* = 4.32; df = 5, 25; *P* = 0.006), and strawberry size (*F* = 5.40; df = 5, 25; *P* = 0.002). Plots receiving abamectin, fenazaquin, pyridaben or spiromesifen had greater yield and larger strawberries than control plots (Fig. 2A, D). Plots receiving abamectin, pyridaben or spiromesifen had more marketable strawberries than control plots (Fig. 2B). Plots receiving abamectin or pyridaben had a lower percentage of cyclamen mite injured strawberries than control plots (Fig. 2C).

## Discussion

Cyclamen mite is a difficult pest to manage due in part to the lack of acaricides available for control. Fenazaquin has been previously tested once against cyclamen mite (Łabanowska 2003), and foliar applications in the greenhouse and field in the experiments in this study reduced mite numbers to similarly low levels as abamectin, the current standard. Pyridaben was also effective, and chlorfenapyr and spiromesifen reduced mite numbers in most cases, although plants treated with the latter usually had more mites numerically than plants treated with other acaricides. In the field all acaricides improved yield and decreased fruit injury due to cyclamen mite. Biopesticide dips of infested transplants had no effect on cyclamen mite numbers, yield or fruit injury levels.

Unlike abamectin, fenazaquin is not translaminar, and thus its efficacy, like the other tested acaricides, is dependent on application considerations, mainly surfactants and water volume. A non-ionic surfactant, such as Agral® 90, has a low surface tension that promotes spreading activity of active ingredients over plant surfaces and into stomata (Stevens 1993; Bonnington et al. 2004). Surfactants can reduce active ingredient amounts (eg., Riga et al. 2006), although strawberry has comparably waxy leaves that may limit surfactant-enabled spreading effects (Cowles et al. 2000). However, adding surfactants improved efficacy of most acaricides against cyclamen mite in strawberry (Fountain et al. 2010; Łabanowska et al. 2015) and is recommended with fenazaquin applications.

Applications in high water volumes (750–1000 L/ha) (Fountain et al. 2010; Łabanowska et al. 2015) have been used to drench strawberry plants and deliver acaricides into plant crowns and new, folded leaves. Results from the greenhouse experiments showed that 2 mL of water per plant was inadequate for reducing cyclamen mite in the crowns, with the exception of fenazaquin in the second experiment. Lower water volume applications may be effective against the proportion of cyclamen mite in the new leaves, but if mites in the crowns are not killed, then populations will rebound and injury to new leaves will occur. Proportions may vary depending on population levels, as at low levels (greenhouse experiment 1) there were about equal numbers of cyclamen mite per leaf and crown, but at high levels (greenhouse experiment 2) there were 4X more mites per leaf than crown. Achieving up to 1000 L/ha can be difficult with tractor-mounted sprayer equipment, but driving speed, nozzle selection and weather forecast should be considered (Deveau 2024). Future research on optimizing water volumes and surfactant rates using grower-scale equipment is needed to maximize the effect of using fenazaquin and other acaricides against cyclamen mite at varying population levels.

Spiromesifen was unique among the acaricides tested because it did not reduce cyclamen mite numbers to as low levels as other acaricides, and in the second greenhouse experiment plant injury increased after application. However, at 2 and 6 weeks after application in the field, the number of cyclamen mite with spiromesifen plots was comparable to other acaricides and significantly less than in the control plots. Spiromesifen is primarily active against juvenile life stages and is also ovicidal, impacting lipid biosynthesis (Nauen et al. 2003). Zalom et al. (2009) found 35% survival of motile cyclamen mite on spiromesifen-treated leaf discs after 48 h, but the proportion of juvenile to adult motiles was not specified. At two weeks after application in the second greenhouse experiment, there was about 50% fewer mites on spiromesifen-treated plants compared to the control, but in the field the reduction at two weeks was 94%, indicating better efficacy in field conditions. In the week following application, during which most spiromesifen residues dissipate (Sardar et al. 2022), there was 21.2 mm of rainfall (ECCC 2024) that may have washed spiromesifen residues into new leaves and crowns of the plants, whereas in the greenhouse irrigation was applied only to the potting mix around the plants. Overall, spiromesifen, like spirotetramat, also a Group 23 mode of action (IRAC 2024), has moderate effects on cyclamen mite (Łabanowska et al. 2015) and could be used in rotation with other acaricides to maintain low population levels.

Acaricidal effects on cyclamen mite translated to measurable improvements in yield in the following season, almost 1 year after application. Cyclamen mite numbers were low 6 weeks after application, likely remained low during autumn, and then were further reduced by winter conditions (Renkema et al. 2022). Even though cyclamen mite numbers were close to or greater than nominal thresholds less than a week before harvesting started in spring 2023 (Government of Alberta 2008; Burrack and Teonnisson 2014), injury levels to fruit were still quite low (0.5 to less than 2%), and strawberry weight of about 9 g averaged over the harvest season is near normal for ‘Jewel’ (Hughes et al. 2013). A spring acaricide application could be made or predatory mites could be introduced to prevent cyclamen mite populations from increasing (Tuovinen and Lindqvist 2010). However, for predatory mites release timing is critical, as species such as *Neoseiulus cucumeris* are cold sensitive and can likely only access cyclamen mite in new leaves once they have moved up from overwintering in the crowns (Patenaude et al. 2020).

Dipping bareroot strawberry transplants in oil and soap treatments for 30 s was not an effective method for reducing cyclamen mite. The number of cyclamen mite 3 months after planting ranged from 18 to 31 per leaf across the treatments, higher than nominal thresholds. The better efficacy of oils and soaps against thrips and whiteflies on vegetative floral cuttings for propagation (Buitenhuis et al. 2016, 2019) may simply be due to the fact that cyclamen mite are concealed and protected in the crown of strawberry transplants, whereas thrips and whiteflies are exposed on leaf surfaces and more easily washed off or affected by the product. Longer dipping times and/or more agitation of transplants in products could improve results. Haack et al. (2018) dipped strawberry transplants for 4 min in a biofungicide for anthracnose control. However, longer transplant exposure to some oils may result in unacceptable levels of phytotoxicity, as in this experiment Landscape Oil and Captiva Prime caused 8–9% plant loss compared to 0–4% for other treatments and the control. Future research on longer dip times for cyclamen mite control, considering active ingredient concentrations in products, application rates and exposure times on plant survival and growth may be warranted.

In conclusion, pre-plant cyclamen mite control on strawberry transplants with dips in oils and soaps is not recommended. Even though pre-plant control methods like steam treatment require investment in infrastructure, they are highly efficacious against cyclamen mite and also control pathogens or nematodes that may be on transplants (Khanal et al. 2020; Johansen et al. 2022; Gahatraj et al. 2023). Once transplants are established in a field and cyclamen mite control is needed, fenazaquin (Magister® SC) is an effective alternative to abamectin (Agri-Mek® SC). Future research is needed to evaluate rotations of both acaricides, particularly with regard to application timing in relation to cyclamen mite populations levels and strawberry plant size. For example, abamectin may be more suitable for spring application in short-day cultivars due to its translaminar capabilities, and fenazaquin could be used post-renovation in July when most of the plant leaves have been mowed-off. Finally, growers can expect an effect of pyridaben (Nexter® SC) on cyclamen mite, and to a lesser degree spiromesifen (Oberon™ Flowable), if these products are used against two-spotted spider mite.

## Data Availability

The datasets generated during the current study are available from the corresponding author on reasonable request.
